# *In vivo* biomechanical measurement and haptic simulation of portal placement procedure in shoulder arthroscopic surgery

**DOI:** 10.1371/journal.pone.0193736

**Published:** 2018-03-01

**Authors:** Sanghoon Chae, Sung-Weon Jung, Hyung-Soon Park

**Affiliations:** 1 Graduate School of Medical Science and Engineering, Korea Advanced Institute of Science and Technology (KAIST), Daejeon, South Korea; 2 Department of Orthopaedic surgery, Samsung Changwon Hospital, Sungkyunkwan University School of Medicine, Changwon, South Korea; 3 Department of Mechanical Engineering, Korea Advanced Institute of Science and Technology (KAIST), Daejeon, South Korea; LAAS-CNRS, FRANCE

## Abstract

A survey of 67 experienced orthopedic surgeons indicated that precise portal placement was the most important skill in arthroscopic surgery. However, none of the currently available virtual reality simulators include simulation / training in portal placement, including haptic feedback of the necessary puncture force. This study aimed to: (1) measure the *in vivo* force and stiffness during a portal placement procedure in an actual operating room and (2) implement active haptic simulation of a portal placement procedure using the measured *in vivo* data. We measured the force required for port placement and the stiffness of the joint capsule during portal placement procedures performed by an experienced arthroscopic surgeon. Based on the acquired mechanical property values, we developed a cable-driven active haptic simulator designed to train the portal placement skill and evaluated the validity of the simulated haptics. Ten patients diagnosed with rotator cuff tears were enrolled in this experiment. The maximum peak force and joint capsule stiffness during posterior portal placement procedures were 66.46 (±10.76N) and 2560.82(±252.92) N/m, respectively. We then designed an active haptic simulator using the acquired data. Our cable-driven mechanism structure had a friction force of 3.763 ± 0.341 N, less than 6% of the mean puncture force. Simulator performance was evaluated by comparing the target stiffness and force with the stiffness and force reproduced by the device. R-squared values were 0.998 for puncture force replication and 0.902 for stiffness replication, indicating that the *in vivo* data can be used to implement a realistic haptic simulator.

## Introduction

Since the 1990s, minimally invasive surgery (MIS), such as arthroscopy, endoscopy, and laparoscopy, has become an important surgical modality. In orthopedic surgery, arthroscopic surgery is now the treatment of choice for surgical management of intra-articular lesions [1]. Because minimally invasive surgical techniques make use of small entry portals to approach the lesion, arthroscopy can offer better surgical outcomes in terms of reduced pain, potential complications, lower risk of infection, and more rapid recovery compared to open surgery [2, 3]. Despite these advantages, it is technically difficult for various orthopedic simulators, including synthetic models, virtual reality (VR) simulators, and human cadaveric specimens, to achieve an environment suitable for repetitive and realistic practice of surgical procedures [4]. Synthetic models replicate the anatomical structure with hard plastic and flexible rubber, but cannot be reused and have a limited number of pathologic conditions that can be simulated. VR simulators are technologically advanced and can allow for extensive repetition of the surgical procedure, but they are expensive (USD $110,000 –$167,000) [5]. Human cadaveric specimens are also expensive (USD $930 –$1,510) [1] and are not reusable. However, orthopedic surgeons and residents have previously ranked cadaveric specimens as the best option for practice surgeries, because they offer a high-fidelity simulation model, compared to a VR simulator [1, 6]. The high fidelity of cadaveric specimens is primarily dependent on the realistic simulation environment and the presence of realistic sensory feedback.

Although training with cadaveric specimens is popular, there are several drawbacks. The sensory feedback may be unnaturally rigid, depending on the preparation of the specimen [7] and there are limited opportunities for practice. According to a survey that estimated the number of repetitions necessary to achieve minimal proficiency in MIS, shoulder arthroscopy required at least 23 repetitions for minimum proficiency [8]. Furthermore, the stakes are high and surgeons are expected to perform the procedure perfectly every time they operate on a patient, including the first time they have performed the surgery. Simulation using a cadaveric model and/or the classic Halstead method (i.e., “See one, do one, teach one.”) cannot provide enough opportunities for trainees to become proficient. In addition, the reduction in training hours, public awareness of patient safety, and increased pressure for operating room efficiency [9, 10] have led to an increased need for realistic and repeatable surgical simulators [11].

To date, practice with a laparoscopic VR simulator has been validated to be able to improve performance in the operating room and to shorten the learning curve of surgeons [12, 13]. However, the transfer validity of an arthroscopic VR simulator, (i.e., the direct transferability of skills from virtual simulation to the operating room), has not yet been clarified [14, 15]. To improve the validity of an arthroscopic simulator, we examined criticisms of a currently available arthroscopic simulator. The passive haptic device (VirtaMed AG, Zürich, Switzerland; SKATS, University of Sheffield, Sheffield, England) which uses a synthetic knee model, showed face validity for navigation and triangulation [16], but had low scores in terms of the tactile sensation [17]. Although some VR simulators are equipped with an active haptic force feedback system, there were no VR simulators with realistic haptic feedback. The primary obstacle for implementation of realistic haptic feedback in current simulators is the wide variety of force characteristics intrinsic to many operations and even variability in the forces used within stages of a single operation [18, 19]. Additionally, none of the available VR simulators incorporates training in portal placement, which is the first key skill encountered when performing arthroscopic surgery [1]. Portal placement has been ranked the highest priority skill that residents must learn prior to performing actual surgery [20].

Therefore, in this study, we aimed to enhance a shoulder arthroscopic VR simulator through *in vivo* data measurement during a portal placement procedure. Although the current VR simulators can aid the trainee in navigation and triangulation skills [21, 22], more cost-effective tools, such as box trainers, can also achieve the same goal without the high cost and technology [23]. The VR simulators, which are much more expensive and technologically advanced, must be able to provide a highly realistic environment to the trainee, including simulation of a portal placement procedure. To fulfill this goal, we acquired *in vivo* biomechanical data during an actual portal placement procedure in the operating room and implemented an active haptic VR force feedback system based on the *in vivo* data. Finally, we assessed the validity of the reproduced force and stiffness via laboratory experiments.

## Materials and methods

### Subjects

Ten patients undergoing arthroscopic shoulder surgery participated in the study, which was approved by the Institutional Review Board of Samsung Changwon Medical Center to the rules stated in the Declaration of Helsinki. Written informed consent was obtained from each patient before surgery. This study were conducted between February 2017 and July 2017. All participants (Mean age: 59.1 ± 6.4 y; Age range: 44–65 y; 5 female, 5 male) had a small to medium sized complete rotator cuff tear without frozen shoulder. Passive range of motion (ROM) tests were conducted one day before the surgery to check whether participants had frozen shoulder. The criteria for excluding frozen shoulder was forward elevation less than 120°, external rotation (with the arm at the side) less than 30°, or internal rotation at the back lower than L3 [24]. Tear size was classified by the surgeon using the De Orio and Cofield System [25].

### *In vivo* data acquisition

**Fig 1:**
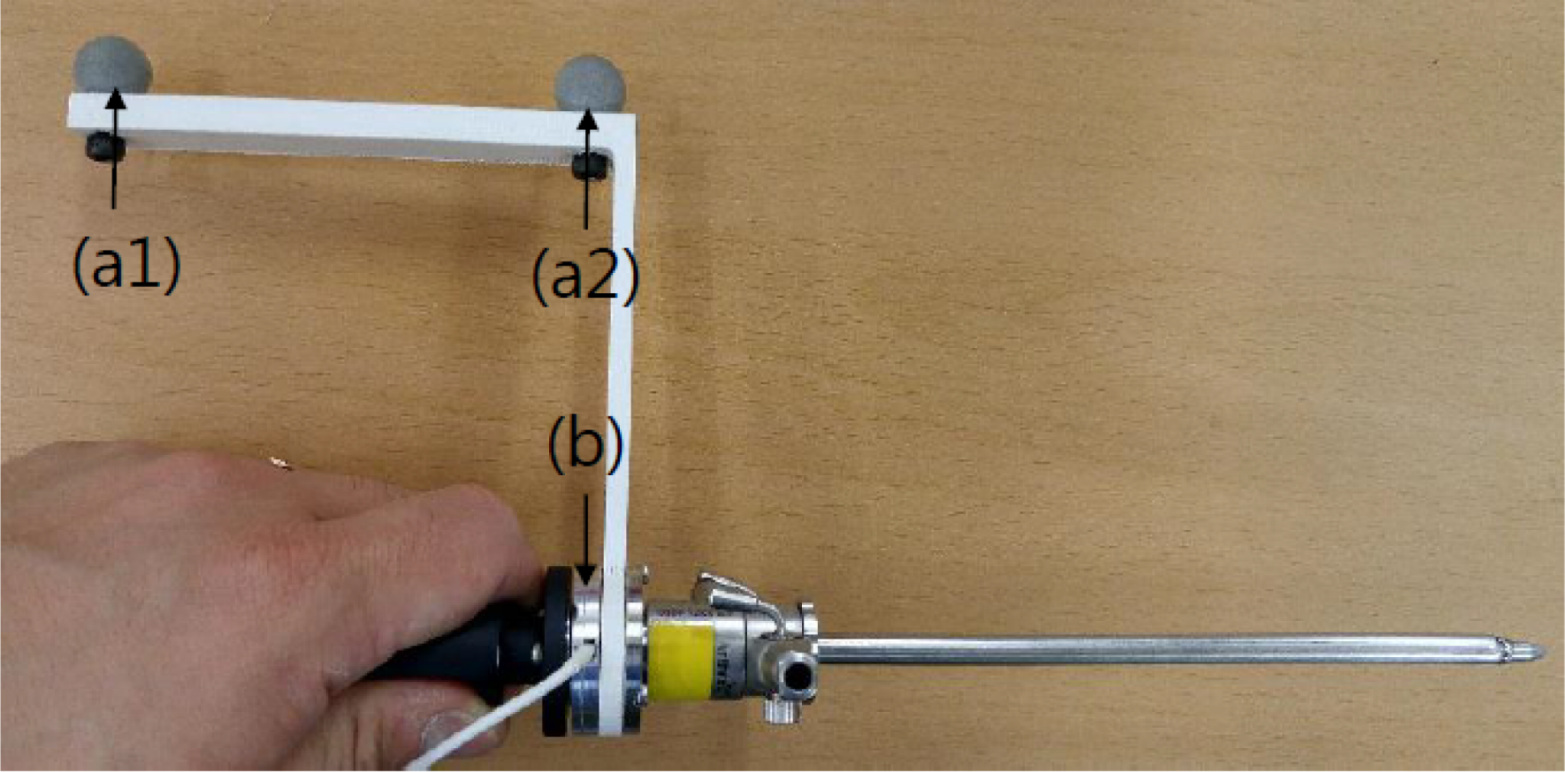
Modified arthroscopic trocar instrument. (a1) Position markers1; (a2) Position markers2; (b) Force sensor.

**Fig 2:**
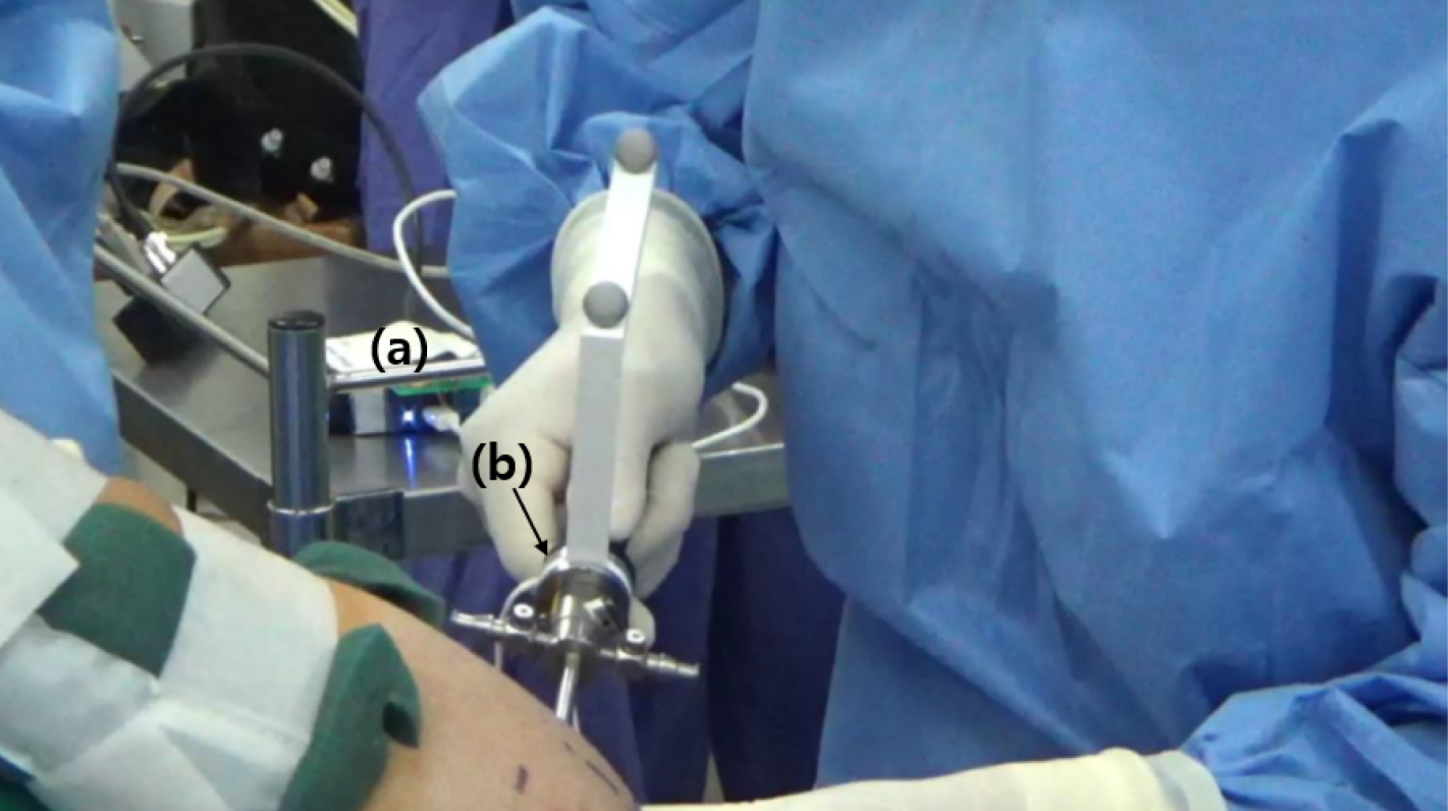
*In vivo* data acquisition procedure. (a) Force transmission module; (b) modified arthroscopic trocar instrument.

All data acquisition was performed by a single surgeon. To acquire the mechanical property data for the force feedback simulator, we modified an arthroscopic trocar (AR-3375-4001, Arthrex, Naples, FL USA) by adding a force sensor and position markers between the handle and shaft of the trocar (Fig 1). A camera (Optitrack, NaturalPoint, Corvalis, OR USA) for detecting marker positions and a data acquisition box (NI-DAQ USB-6002, National Instrument, Austin, TX USA) were installed in the operating room, distant from the surgical field (Fig 2). The accuracy of the force sensor (i2a Systems, Daejeon, South Korea) and portable motion camera was within 1 Newton and 1 mm, respectively. The marker positions and force data were synchronously acquired at a sampling rate of 100 Hz.

**Fig 3:**
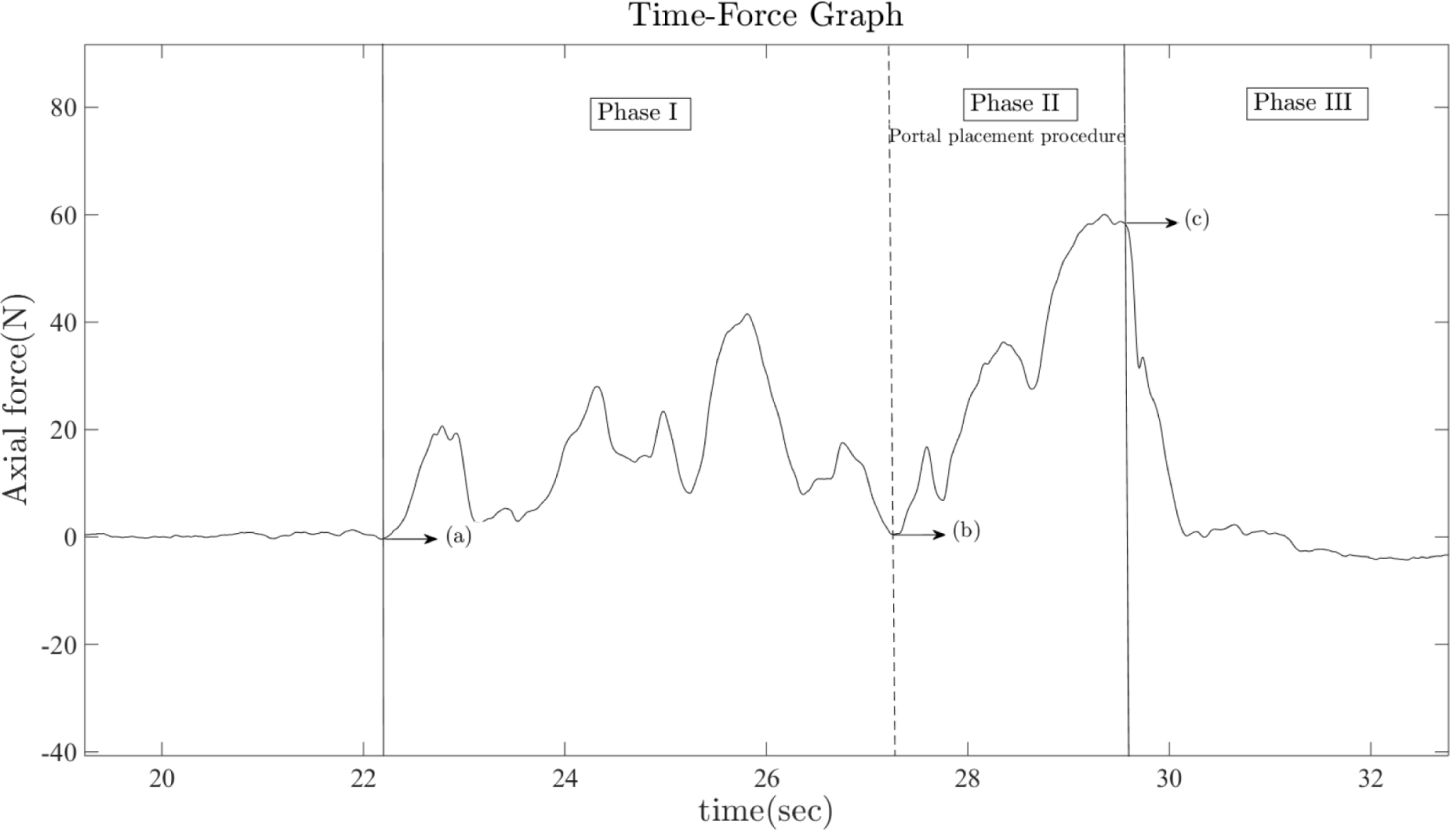
Phase interpretation for measuring stiffness and puncture force. Phase I represents the preparation period for placing the modified trocar on the “soft spot”, which is located between infraspinatus and teres minor muscle.; Phase II represents the interval containing the actual posterior portal placement procedure; Phase III represents the period after portal placement; (a) Trocar insertion point on skin surface; (b) Set point for puncture on soft spot; (c) Puncture point, in which maximum puncture force is acquired.

The surgical procedure investigated was placement of a posterior portal, which is the primary entry portal for shoulder arthroscopy. As soon as the surgeon placed the trocar against the shoulder capsule in the “soft spot”, specifically between the infraspinatus and teres minor muscles, we started capturing force and marker position (trocar displacement). Data collection continued as the surgeon advanced the modified trocar forward into the shoulder capsule, targeting the coracoid process. The total travel distance of the instrument was calculated by the sum of the dot-product of the two vectors: the norm vector along the trocar shaft and the norm vector of the trocar movement. During the surgical procedures, the circulating nurse recorded three times: 1) start of the procedure, 2) puncture time, and 3) end of the procedure based on a comment from the surgeon. These times were used to confirm three phases of portal placement during data analysis (Fig 3).

Patient safety was the most critical part of this experiment. Patient safety was ensured by using a thorough sterilization procedure. All instruments that were near the surgical field were sterilized using ethylene-oxide gas, including the force sensor and position markers. In addition, after completing a single portal placement procedure, no additional attempts at portal placement were made. Thus, if either of the two position markers was lost to view during the surgical procedure, the procedure could not be repeated to obtain valid *in vivo* displacement data. Therefore, while obtaining the *in vivo* stiffness, position data was unable to be obtained for 5 patients. The position marker(s) was hidden for a portion of time during the procedure, either by the body of an assistant or it was covered by the surgeon’s hand, or by having the instrument positioned in such a way that the markers were not visible to the camera during the procedure. After surgery, no patients had any surgical complications related to the experiment.

### Post-processing of the *in vivo* data

After obtaining the *in vivo* force and marker position data, the raw data was low-pass filtered (cut-off frequency: 5 Hz) to remove high-frequency noise. The time course of the force data was divided into three phases (Fig 3), based on the times recorded by the circulating nurse during the portal placement procedure. Phase I is the preparation period for setting the modified trocar to the shoulder joint capsule, Phase II represents the interval that contains the actual portal placement procedure, and Phase III represents the state after puncture. Therefore, the maximum puncture forces during the portal placement procedure were measured from the force values at the end of Phase II. In order to find the stiffness value of the *in vivo* shoulder joint capsule, we used Hooke’s law. This stiffness was replicated with a force feedback system after the experiment, using the same law:

F(t)=kx(t) (1)

Where F, k, and x represent the force applied to the trocar handle, the stiffness value during the portal placement procedure, and displacement of the instrument, respectively. We calculated the joint capsule stiffness for each patient using a least-squares linear regression model.

### Implementation of active haptic force feedback

**Fig 4:**
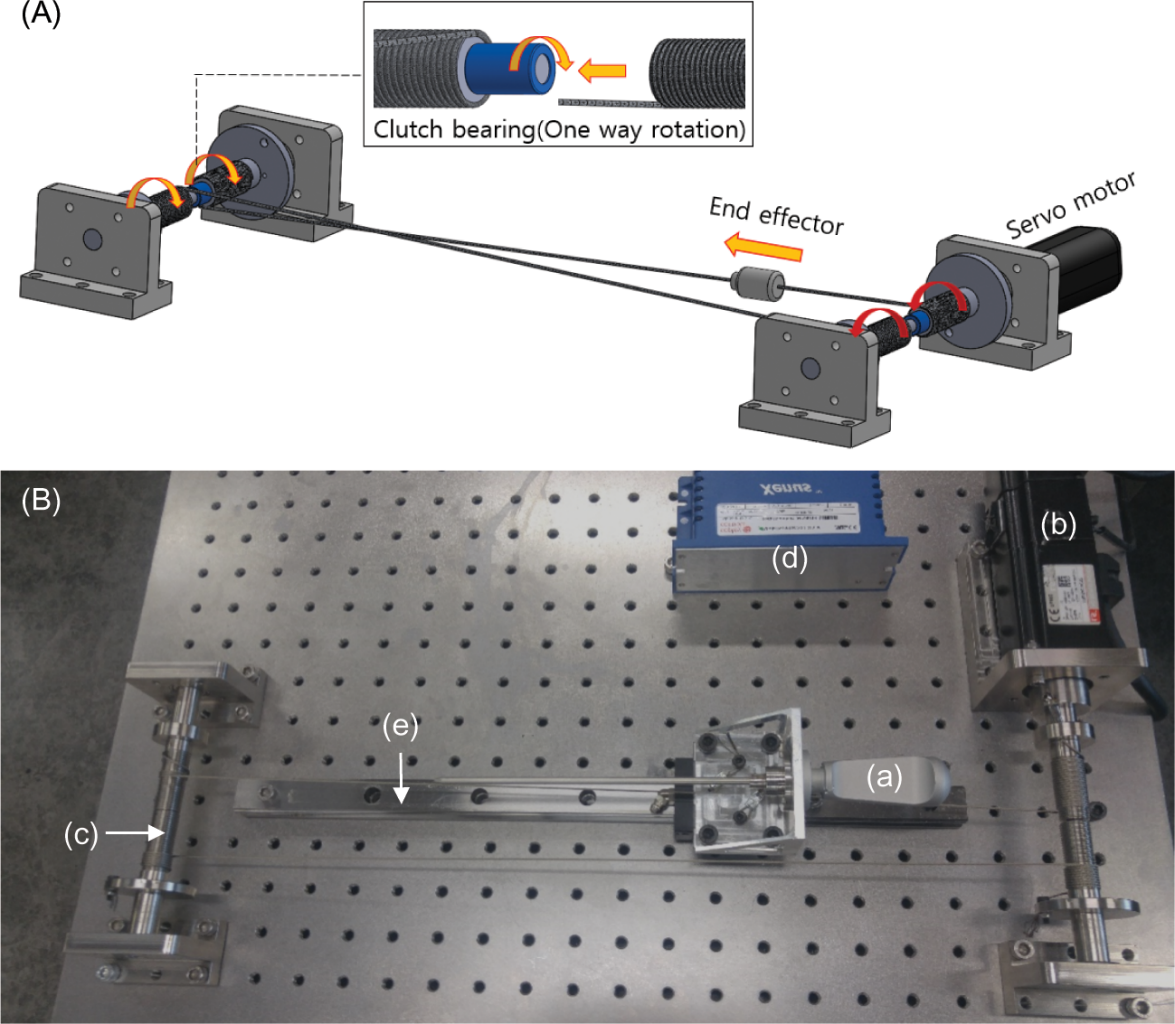
Implementation of active haptic force feedback system. (A) CAD diagram for representing the cable driven mechanism. (B) Haptic force feedback system. (a) end effector; (b) servo-motor; (c) cable reel; (d) motor driver; (e) linear guide.

We have developed an active haptic force feedback system in which the structure is based on a cable mechanism (Fig 4A and 4B). It can create push-and-pull forces with one servo-motor without a cable-sagging problem. This system uses one servo-motor (APM-SA01ACN2, LS Mecapion, Daegu, South Korea), motor-driver (Xenes XSJ-230-06, Copley Controls, Canton, MA USA), wire cable (2047, Carl Stahl Sava Industries, Riverdale, NJ USA), two clutch bearings (BHFL6, MISUMI, Tokyo, Japan), three ball bearings (6900ZZ, MISUMI, Tokyo, Japan), four cable reels, and four mounting structures. Each mounting structure was designed using a 3D CAD program (SolidWorks, Dassault Systèmes, Vélizy-Villacoublay, France) and manufactured using stainless steel. While the user moves the end effector forward, the posterior cable reel rotates in the counterclockwise direction and the anterior cable reel rotates clockwise, because each cable is wound in opposite directions.

**Fig 5:**
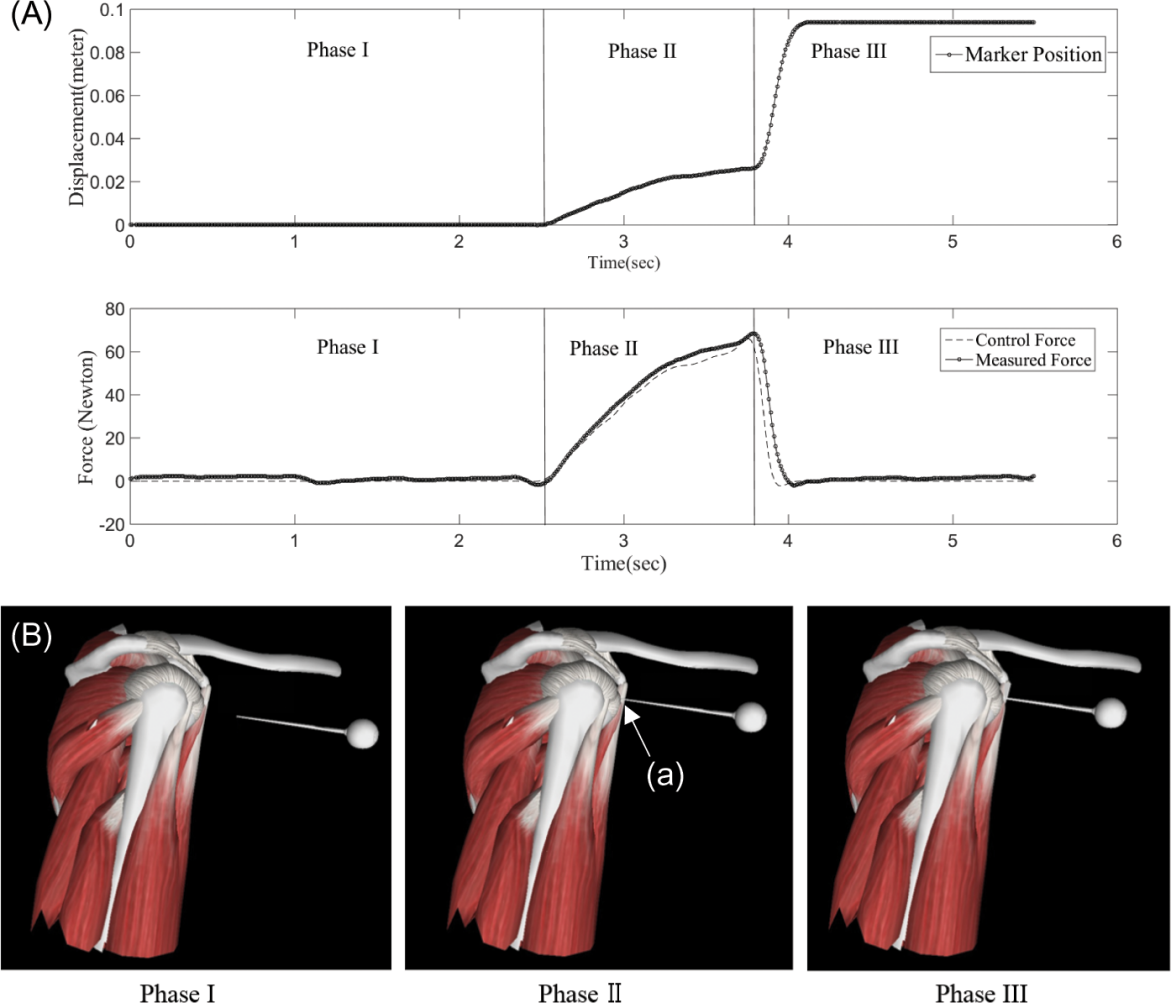
Force feedback synchronized with virtual reality graphic environment. (A) Displacement (upper) and force (lower) graph based on data acquired from haptic device. (B) Virtual reality graphic environment. Phase interpretation of haptic force feedback simulation: Phase I represents the pre-puncture state for the trocar to contact the shoulder joint capsule; Phase II shows the conflict of trocar on soft spot (a); Phase III represents the post-puncture state of portal placement.

In addition, we created a virtual reality (VR) graphic environment for the shoulder joint and trocar instrument using the 3ds Max (Autodesk, USA) and OpenGL programs. The active haptic force feedback system was synchronized with the VR computer graphics engine (Fig 5A and 5B). Any collision between the VR instrument and the shoulder joint capsule was detected by the OpenGL graphics program. It calculated the distance between the trocar tip and the shoulder joint capsule based on graphical position data. The varying position of the trocar was detected by the encoder attached to the motor. When the user pushes the handle of the simulator, the trocar approaches the VR shoulder joint. When the trocar hits the shoulder joint capsule in VR, the simulator motor creates a feedback force based on the distance from the collision point and the *in vivo* stiffness value. Once the force exceeds the maximum peak puncture force for portal placement, the simulator suddenly releases the force to zero. The stiffness is implemented by varying the force according to the travel distance of the trocar. System specifications are detailed in Table 1.

**Table 1:** Haptic force feedback device specification.

Degrees of freedom	1
Workspace	30 cm
Maximum force	137.1 Newtons
Moment of inertia (motor)	0.05 * 10^−4^ *Kg* ∙ *m*^2^
Moment of inertia (cable reel)	34833.32 * 10^−9^ *Kg* ∙ *m*^2^
Encoder	2,048 pulse/Rev
VR data transmission rate (Hz)	100 Hz
Materials	Stainless steel

### Evaluation of the active haptic force feedback system

To evaluate the haptic system, we carried out two laboratory experiments. First, in the haptic simulator, minimizing friction force is important since friction force causes error between the targeted force feedback and the real force felt by the user. To assess the magnitude of the friction force in the haptic simulator, we attached the same force sensor used for *in vivo* data acquisition to the motor and evaluated the displacement using the encoder also attached to the motor. The experiment was conducted ten times by pushing the end-effector swiftly with the cable attached to the handle. Afterward, the same experiment was carried out with the cable detached from the handle to measure the friction without the cable. According to the Law of Conservation of Energy, we assumed that all of the kinetic energy in the simulator handle and the rotational energy in the cable reel is dissipated by the friction energy. Therefore, by subtracting the two friction forces, we calculated the friction force contributed by the cable alone.

Second, to evaluate the realism of the active haptic force feedback system, we compared the stiffness and force simulated by the haptic device with the *in vivo* stiffness and force values. The force and displacement graphs over time during a simulated portal placement procedure are shown in Fig 5A. In Fig 5B, the control force represents the desired force that the trainee should feel and the measured force represents the actual force the trainee felt, measured using the force-sensor attached to the handle. The displacement data were measured by the encoder attached to the motor. With these experimental data, we measured the R-squared values of force and stiffness replication as validation of the simulation.

## Results and discussion

**Fig 6:**
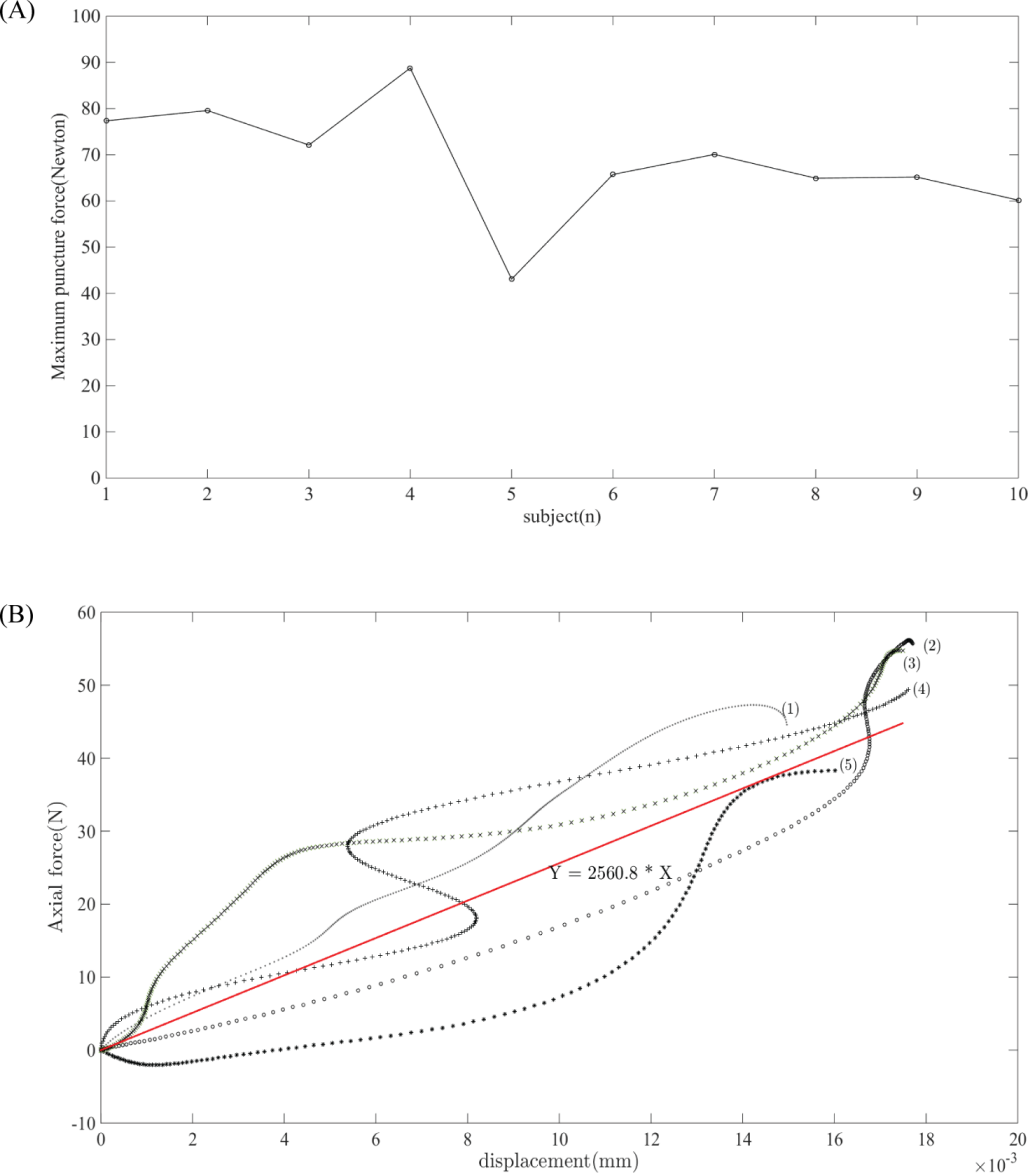
Visualization of acquired *in vivo* data. (A) Maximum puncture force of ten patients during posterior portal placement procedure. (B) Force-displacement plot of five patients for calculation of stiffness; (1)–(5) represents force-displacement plot of five subjects; the solid red line represents mean stiffness.

Maximum puncture forces for all ten patients are shown in Fig 6A. Fig 6B demonstrates the *in vivo* force-displacement graph for five patients and the mean stiffness for the same five patients during Phase II. The *in vivo* mechanical properties obtained during an arthroscopic portal placement procedure are shown in Table 2. The first row shows the means and standard deviations for puncture force and stiffness, which were used as the default values for the haptic force feedback simulation. The second row shows the range of the *in vivo* biomechanical data obtained from ten subjects. For evaluation of the haptic force feedback, the range of puncture force and stiffness were implemented on the haptic device so that the trainee could experience variable patient conditions.

**Table 2:** Mechanical properties of the portal placement procedure*.

	Puncture Force (N)	Stiffness (N/m)
Mean (±Std.)	66.46 (±10.76)	2560.82 (±252.92)
Range of *in vivo* data	43.08–79.58	2301.04–2889.70

* One patient data (Patient 4 in S1 Table) was excluded in calculation of mean (±Std.) and range of *in vivo* data since clear evidence of frozen shoulder (severe synovitis) was found in the arthroscopic image.

Prior to implementing the mechanical properties (S1 Table) on the haptic simulator, we evaluated the performance of the haptic device itself. To evaluate the accuracy of the target force as implemented using the haptic device, the friction force inherent in the device was measured. The entire friction force was 3.76 (±0.341) N and the friction force of the linear guide was 1.91 (±0.12) N. The friction force caused by the cable mechanism was 1.85 (±0.316) N. The entire friction force, which could cause a force-control error, was less than 6% of the mean puncture force and was smaller than the JND (just noticeable difference) of human perception [26].

**Fig 7:**
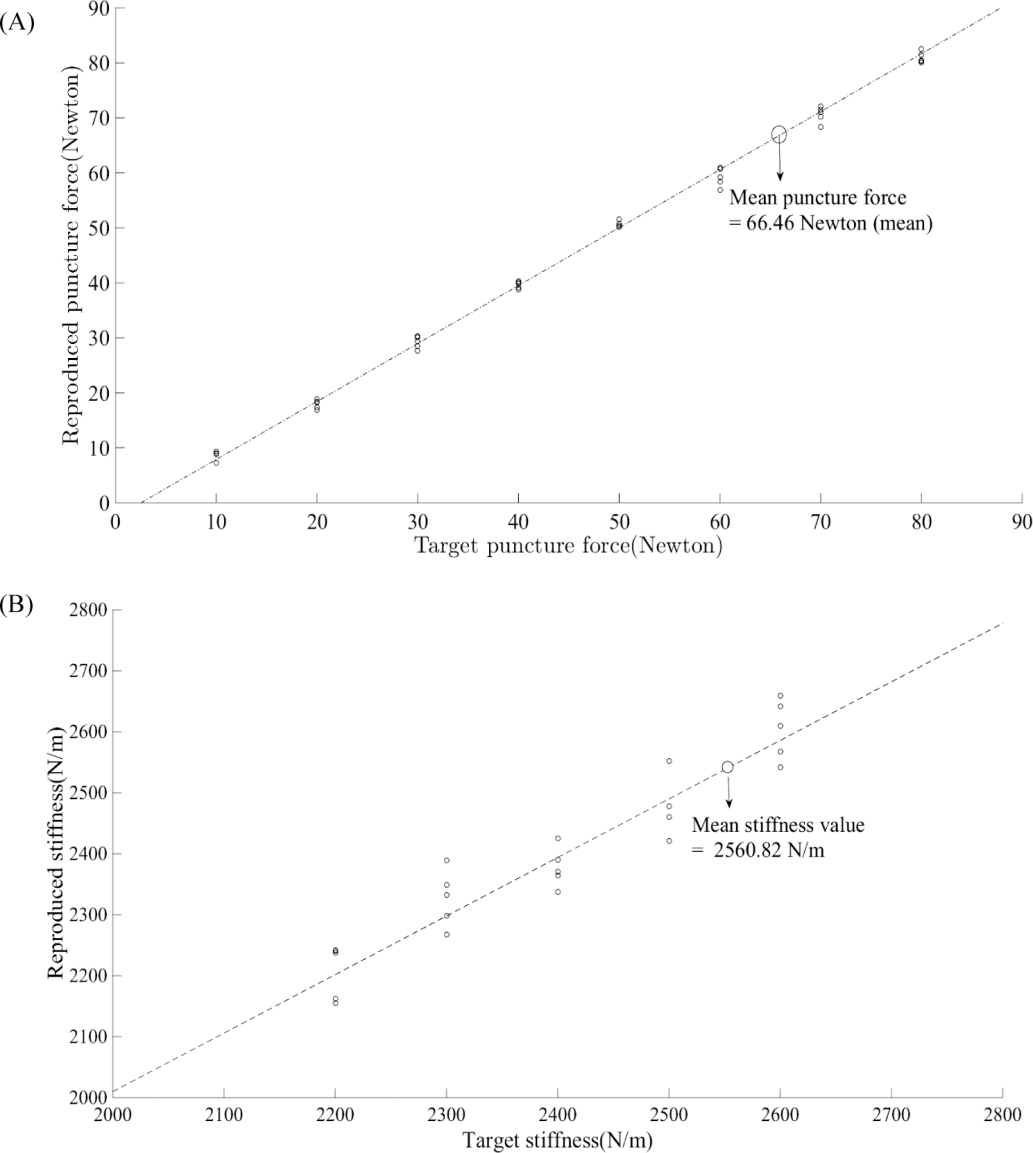
Validation of haptic force feedback system. (A) Target vs. reproduced puncture force using the haptic device. (B) Target vs. reproduced stiffness using the haptic device.

As an evaluation of the system, we also verified the reproduced force and stiffness during the simulated portal placement procedure. Fig 7A and 7B show the reproduced force and stiffness with respect to the target puncture force and stiffness, respectively. R-squared values of the replicated force and stiffness were 0.998 and 0.902, respectively.

This study focused on the ‘portal placement procedure’ which has been ranked as the first priority of an arthroscopic simulator. Although no existing simulators have implemented this procedure, haptic simulation of portal placement is one of the essential training steps for trainees, because the iatrogenic nerve or cartilage damage that can occur with inappropriate puncture force are common and dangerous complications of arthroscopic surgery [27, 28]. Our study is the first approach to measure simulation parameters via *in vivo* data acquisition in the operating room and to use those parameters for a haptic simulator.

Realistic force feedback should be based on actual *in vivo* values. However, few studies have reported the *in vivo* mechanical properties of biologic tissues. For example, previously published studies have reported the *in vivo* mechanical properties of porcine liver and stomach and used a parallelogram-type haptic device (PHANTOM Premium-T, SensAble Technologies, Inc., Woburn, MA USA) for force and displacement measurement and gave a ramp-and-hold stimulus to obtain stiffness values while varying the indentation depth and stimulation frequency [29, 30]. However, during actual surgery, this method is impractical, as it is prohibitively difficult to use additional mechanical devices and to try various indentation forces during real surgical procedures. Therefore, we modified an existing surgical instrument for force measurement and used reflective markers for displacement measurement to allow for minimal variation from actual surgical techniques. This enabled the sterilization of experimental instruments and *in vivo* data collection during an actual operation.

We examined the *in vivo* maximum puncture force during actual portal placement procedures. The maximum puncture force is a critical parameter for determining when to release the force rapidly during haptic simulation. As a similar approach, Poddler et al. reported the maximum *in vivo* puncture force of the prostate capsule with an 18-gauge needle as 5.0 N [31]. Considering that the force used during portal placement is much greater (68.8 N), it would be more difficult to immediately release the puncture force during an actual surgical procedure. For example, a novice trainee who has not previously felt the real force or stiffness of the shoulder joint capsule during surgery could use too much or too little force, risking complications for patients [32]. Therefore, the *in vivo* measurement of the maximum puncture force plays an important role in haptic simulation. In addition, we measured the stiffness value during the portal placement procedure. Specifically, the stiffness was measured along the direction from the “soft spot” to the coracoid process which is neither perpendicular nor parallel to the joint capsule. However, in the related literature, it is difficult to find stiffness values specific to this procedure. For example, Bey et al. has reported Young’s modulus of the middle posterior capsule of the glenohumeral joint as 44.9 MPa [33]. This measurement cannot be used for the portal placement procedure for the following reasons. (1) The portal placement procedure not only involves the shoulder joint capsule but also the other soft tissues (i.e, the fascia and connective tissues between the rotator cuff muscles). In our experiment, we measured the force and displacement under the deltoid from the “soft spot,” located between the teres minor and infraspinatus muscles, to the puncture point of the joint capsule. (2) The angle at which the shoulder joint capsule is punctured can vary and is often not perpendicular to the shoulder joint, because the surgeon inserts the trocar aimed toward the coracoid process. Therefore, the current stiffness result will be more suitable for implementing realistic portal placement simulation. In addition, the *in viv*o stiffness measured from our experiment can be used as a reference when creating a realistic model of the shoulder joint including soft tissues and the joint capsule.

This study includes several limitations. First, the *in vivo* data measured in this study is limited to patients with isolated rotator cuff tears. For patients with other combined pathologies, such as a frozen shoulder, the *in vivo* forces may differ dramatically. However, the methods for measurement of *in vivo* data and subsequent implementation with haptic simulation described in this paper can be extended to include additional pathologies. Second, the haptic simulator designed in this study was developed to reflect force in a single degree of freedom (DOF). More realistic simulation environments could be created by implementing a full six DOFs (3 DOFs for instrument position and 3 DOFs for instrument orientation). Fortunately, currently available VR simulators, such as the Arthro Mentor (3D Systems, Airport City, Israel) and the ArthroSim (Touch of Life Technologies (ToLTech), Aurora, CO USA) allow for 6 DOF placement of arthroscopic instruments in three dimensional space. Therefore, we chose to focus on implementing haptic force feedback and the novel implementation of the portal placement procedure, which typically involves linear motion. Third, the stiffness data could only be obtained from five subjects in the current study, due to limitations related to motion capture in the operating room environment. Patient care and sterilization considerations limited our ability to collect duplicate trials on the same subjects. However, we chose to accept these practical limitations, given the added value of obtaining data from actual arthroscopic procedures. Fourth, the passive ROM test could not perfectly exclude frozen shoulder verified by arthroscopic images. Prior to the surgery, the patients with frozen shoulder were excluded according to the criteria based on passive ROM [24]. However, one patient who did not show frozen shoulder by the passive ROM criteria clearly showed frozen shoulder determined by clear synovitis in the arthroscopic image (patient 4 in S1 Table) and two might have frozen shoulder according to the mild hyperemia in the image (patient number 2 and 8).

Proficiency at arthroscopic surgery is reached through repetition and practice. In this era of increasing awareness of patient safety concerns and duty hour limitations, surgeons-in-training must find alternate methods for obtaining the required number of repetitions to reach competence. Although currently available simulators for orthopedic surgery lag behind those available to other specialties [34], this study enhances simulations of arthroscopic shoulder surgery by adding an essential element, a simulated portal placement procedure, and by incorporating force feedback based on in vivo data, which should contribute to improving next-generation active haptic arthroscopic VR simulators.

## Conclusions

In previous eras, the training of an orthopedic surgeon mostly depended on the apprenticeship model. However in the modern era, due to concerns over duty hours and increased awareness of patient safety issues, the training environment is moving away from the traditional apprenticeship model [35, 36]. Particularly due to limitations on duty hours, modern training environments must rely on computer simulation (e.g., VR simulators) as essential training tools. This study enhances currently available simulations of arthroscopic shoulder surgery by adding an essential element, a simulated portal placement procedure, and by incorporating force feedback based on *in vivo* data.

## Supporting information

S1 Table Patient characteristics and acquired *in vivo* data. (XLSX)
